# Cardiovascular Effects of Caffeic Acid and Its Derivatives: A Comprehensive Review

**DOI:** 10.3389/fphys.2020.595516

**Published:** 2020-11-27

**Authors:** Henrique Silva, Nuno Miguel F. Lopes

**Affiliations:** ^1^CBIOS – Universidade Lusófona’s Research Center for Biosciences and Health Technologies, Lisboa, Portugal; ^2^Department of Pharmacological Sciences, Faculty of Pharmacy, Universidade de Lisboa, Lisbon, Portugal

**Keywords:** caffeic acid derivatives, vasorelaxant, blood pressure, antioxidants, cardiovascular protection

## Abstract

Caffeic acid (CA) and its phenethyl ester (CAPE) are naturally occurring hydroxycinnamic acids with an interesting array of biological activities; e.g., antioxidant, anti-inflammatory, antimicrobial and cytostatic. More recently, several synthetic analogs have also shown similar properties, and some with the advantage of added stability. The actions of these compounds on the cardiovascular system have not been thoroughly explored despite presenting an interesting potential. Indeed the mechanisms underlying the vascular effects of these compounds particularly need clarifying. The aim of this paper is to provide a comprehensive and up-to-date review on current knowledge about CA and its derivatives in the cardiovascular system. Caffeic acid, CAPE and the synthetic caffeic acid phenethyl amide (CAPA) exhibit vasorelaxant activity by acting on the endothelial and vascular smooth muscle cells. Vasorelaxant mechanisms include the increased endothelial NO secretion, modulation of calcium and potassium channels, and modulation of adrenergic receptors. Together with a negative chronotropic effect, vasorelaxant activity contributes to lower blood pressure, as several preclinical studies show. Their antioxidant, anti-inflammatory and anti-angiogenic properties contribute to an important anti-atherosclerotic effect, and protect tissues against ischemia/reperfusion injuries and the cellular dysfunction caused by different physico-chemical agents. There is an obvious shortage of *in vivo* studies to further explore these compounds’ potential in vascular physiology. Nevertheless, their favorable pharmacokinetic profile and overall lack of toxicity make these compounds suitable for clinical studies.

## Introduction

Caffeic acid (CA) is a hydroxycinnamic acid that belongs to the phenolic acid family of polyphenols. It is the main hydroxycinnamic acid present in the human diet, with the highest content being found in blueberries, kiwis, plums, cherries, and apples, although also present in cereals, carrots, salad, eggplants, cabbage, artichoke, and coffee (El-Seedi et al., 2012; Del Rio et al., 2013; Sova and Saso, 2020). Besides foodstuffs, CA is also present in propolis, a resinous mixture created by honeybees from different botanical sources and a natural product that has been used in folk medicine for many centuries (Silva et al., 2014). Caffeic acid phenethyl ester (CAPE) is a naturally occurring derivative of CA and is also prevalent in propolis, mainly in the poplar variety, made from resinous exudates of buds and young leaves of different *Populus* species, especially *Populus nigra* L. (Bankova et al., 2018). CAPE is the single and most widely studied of all the individual constituents of propolis and is currently thought responsible for most of the poplar variety’s strong antioxidant activity (Russo et al., 2002; Bankova et al., 2018). Besides *Populus* species, CAPE has been identified only in a few other botanical sources (Bankova et al., 2018). In the last few decades, much interest has been shown in exploring the biological properties of CA, CAPE, and closely related derivatives, mainly synthetic ones. These compounds are known to display antioxidant (Sud’ina et al., 1993; Russo et al., 2002), anti-inflammatory (Jung et al., 2008; Doiron et al., 2017), immunomodulatory (Natarajan et al., 1996), cytostatic (Jung et al., 2007), antibacterial (Arasoglu et al., 2016), and antiviral (Erdemli et al., 2015) properties. Besides these important activities, CA and its derivatives have shown a very high potential for treating and preventing cardiovascular diseases in preclinical studies. Their useful biological activities, coupled with favorable safety profiles, make them good candidates for clinical studies. The aim of this paper is to provide a comprehensive and up-to-date review of the main cardiovascular actions attributed to CA and its derivatives both *in vitro* and *in vivo* in order to highlight their main therapeutic potentials.

## General Characterization, Bioavailability, and Metabolism

Caffeic acid [(E)-3-(3,4-dihydroxyphenyl)prop-2-enoic acid] is a white amorphous powder with a molecular mass of 180.16 g/mol. Its partition coefficient (log*P*) differs between publications, ranging between 1.0 and 1.3 (Wu W.M. et al., 2007; Kudugunti et al., 2010; Paracatu et al., 2014). In its free form, CA is absorbed in the gastrointestinal tract through monocarboxylic acid transporters (Konishi and Kobayashi, 2004; Konishi et al., 2004) and, to a lesser extent, through transepithelial flux (Konishi et al., 2003). Gut microbiota is also involved in the metabolism of CA. In fact, under anaerobic conditions, CA undergoes decarboxylation, which is carried out by the bacteria possessing tyrosine decarboxylase, with the corresponding product [3-(3-hydroxyphenyl)-propionic acid] displaying greater antioxidant activity compared to CA (Shen et al., 2020). Once absorbed, CA undergoes extensive metabolic transformations in the liver and kidneys (Ito et al., 2005; Lafay et al., 2006; El-Seedi et al., 2012). Caffeic acid has revealed a good safety profile in a phase 1 clinical trial (NCT02050334) (Information, 2015).

Caffeic acid phenethyl ester [2-phenylethyl (E)-3-(3,4-dihydroxyphenyl)prop-2-enoate] is a white crystalline solid with a molecular mass of 284.31 g/mol. It is lipophilic with logP values differing between publications, ranging between 3.2 and 13.8 (Koltuksuz et al., 1999; Wu W.M. et al., 2007; Paracatu et al., 2014), which allows it to cross the blood-brain barrier in rats (Wei et al., 2004; Barros Silva et al., 2013). As the isolation and purification of CAPE from natural sources are expensive, low-yielding and time-consuming, several chemical and enzymatic synthetic methods have been developed (Bankova et al., 2018). CAPE has been shown to have very low toxicity to normal cells compared to cancer cells (Li et al., 2016). It is easily absorbed after intraperitoneal injection (Koltuksuz et al., 1999). In rat plasma, CAPE is known to remain stable for only 6 h, after which it is metabolized and excreted mostly in urine as both a glucuronide conjugate and unmodified (Wang X. et al., 2007). Given that only a low percentage of the administered dose is recovered, CAPE is thought to be highly susceptible to hydrolysis in plasma and cell-containing esterase enzymes in rats, with CA being the resulting product (Celli et al., 2004; Tang and Sojinu, 2012). This susceptibility is not seen *in vitro* in human plasma, where CAPE is known to be fairly stable by forming a complex with albumin (Celli et al., 2007; Li et al., 2016). The absence of carboxylesterase in human plasma may also contribute to this stability (Celli et al., 2004; Li et al., 2016). CAPE is known to be bioactivated by tyrosinase, an enzyme that is over-expressed in melanoma cell lines, to CAPE-quinone. This compound, like CAPE itself, then reacts with glutathione (GSH) to form the CAPE-SG conjugate. Both the CAPE-SG conjugate and CAPE-quinone inhibit glutathione S-transferase (GST) (Kudugunti et al., 2011). CAPE also interacts with liver cytochrome P450 (CYP) enzymes by decreasing the activity of isoforms CYP1A1/2 and CYP2B1/2 12 h after administration (Beltrán-Ramírez et al., 2008). The pharmacokinetic profile of CAPE was obtained in Sprague-Dawley rats, with a volume of distribution ranging from 1555 to 5209 mL/kg, which lowers with increasing doses. The elimination half-life ranges from 21.2 to 26.7 min and is dose-independent (Wang X. et al., 2009).

**FIGURE 1:**
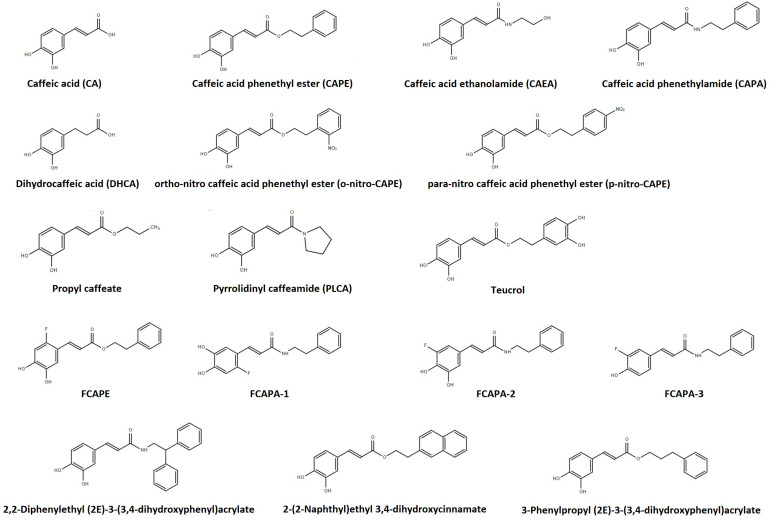
Structure of caffeic acid and its derivatives. This figure was made with ChemSpider software.

The high cost associated with the production of high purity CAPE, which includes using ethanol or dimethyl sulfoxide (DMSO) as solvents, is an obvious obstacle for its clinical use (Yeǧin et al., 2020). Furthermore, given CAPE’s instability in rat plasma and in circulation, several CA and CAPE metabolites and synthetic structural analog compounds have been investigated (see Figure 1). Caffeic acid phenethyl amide (CAPA) is a synthetic derivative that has proven more resistant to esterase hydrolysis for its amide moiety by displaying a 77-fold increase in stability in plasma in relation to CAPE at 37°C (Yang J. et al., 2012). Caffeic acid phenethyl amide is less toxic to normal cells compared to CAPE, although no difference in cytoprotection has been found between the two compounds (Yang J. et al., 2010). Caffeic acid ethanolamine (CAEA) is another synthetic derivative which has, to date, been discussed in only one publication, with no mention to its chemical stability (Lee et al., 2015b). Several CAPE and CAPA fluorinated compounds have also been synthesized and their stability studied (Wang X. et al., 2006, 2007; Yang J. et al., 2010). One of these compounds, 3-(2-fluoro-4,5-dihydroxyphenyl)-acrylic acid phenethyl ester (FCAPE), appears to be more stable than CAPE (Wang X. et al., 2006, 2007), displays cytoprotective action, and its pharmacokinetic profile is also known. Its volume of distribution is 7596 mL/kg and its elimination half-life is 31.6 min (Wang X. et al., 2009). Three fluorinated CAPA derivatives (FCAPA1-3) have also been found to not be toxic and display cytoprotective action (Yang J. et al., 2010). *Ortho-* and *para*-nitro-CAPE derivatives have also been synthetized and display cytoprotective action as well (Du et al., 2016; Li et al., 2018). Several other CA and CAPE derivatives, including alkyl esters, have been investigated, although cytoprotection and stability profiles have only been established for a small portion (Gaglione et al., 2013; Teixeira et al., 2013; Bhullar et al., 2014; Zhang P. et al., 2014; Khan et al., 2016). Only the compounds that show promising biological effects at the cardiovascular system level will be discussed in this paper.

The interest in CA and its derivatives in recent years has lied more in their antioxidant, anti-inflammatory and immunomodulatory properties, while the characterization of their cardiovascular actions has not advanced at the same speed. This paper intends to provide an up-to-date and comprehensive review of the actions of CA and its derivatives in the cardiovascular system, while focusing on their vasoactive actions.

## Biological Activities on the Cardiovascular System

### Vasorelaxant Effect

**FIGURE 2:**
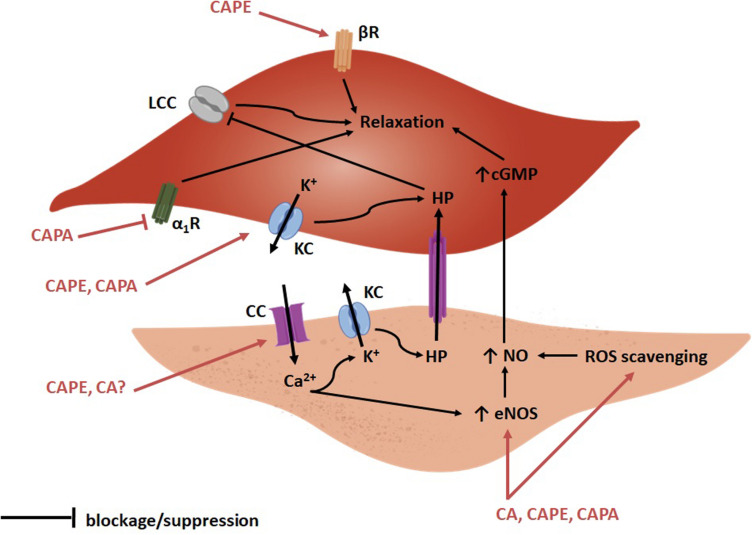
Scheme of the proposed vasorelaxant actions of CA, CAPE and CAPA according to current research. On VSM cells (top) CAPE and CAPA may act on potassium channels (KC), which would lead to potassium efflux and hyperpolarization (HP). Hyperpolarization would contribute to inactivate L-type calcium channels (LCC) and to prevent VSM cell contraction. Additionally, CAPE may activate the beta adrenergic receptor (βR) and contribute to VSM cell relaxation. Additionally, CAPA may exert a weak alpha-1 receptor (α_1_R) blocking effect. On endothelial cells (bottom), CA and CAPE may act on calcium channels (CC) and lead to calcium influx which, in turn, would open potassium channels (KC) and lead to HP. Hyperpolarization could be communicated to VSM cells via gap junctions and reinforce its relaxation. Calcium may also increase endothelial nitric oxide synthase (eNOS) activity and increase NO synthesis, which would diffuse into and relax VSM cells. In addition, CA, CAPE, and CAPA may also scavenge reactive oxygen species (ROS) and prevent NO removal therefore increasing its cellular content.

CA, CAPE, and CAPA all possess vasorelaxant activities by acting mainly on endothelial and vascular smooth muscle (VSM) cells through different molecular targets. Detailed results of papers published on these compounds’ vasorelaxant effects are presented in Table 1. Figure 2 provides details of the currently proposed mechanisms for the vasorelaxant action of CA and its derivatives in the endothelial and VSM cells. The studies that have assessed the vascular action of these compounds have used norepinephrine (NE), phenylephrine (PE), potassium or potassium chloride (KCl), prostaglandin F2_α_ (PGF2_α_) and endothelin (ET) as pre-constricting drugs in blood vessel rings that are either intact or denuded (i.e., with no endothelium). In a medium provided with calcium, NE, PE, PGF2_α_, and ET induce VSM cell contraction through the receptor-mediated activation of phospholipase C and inositol triphosphate/diacylglycerol pathway, which leads to calcium being released from intracellular stores (Watanabe et al., 1994; Malhotra, 1998; Cotecchia, 2010; Horinouchi et al., 2013). Potassium and KCl-induced contraction are due to membrane depolarization, which results in calcium influx through voltage-gated channels (Godfraind et al., 1986).

**TABLE 1:** Description and main results of the studies (*in vitro*, *ex vivo*) characterizing the effect of caffeic acid and its derivatives on the vasculature (CA–caffeic acid; CAPE–caffeic acid phenethyl ester; CAPA-caffeic acid phenethyl amide; w.o.–weeks old; m.o.– months old).

Authors	Species/strain (age, weight)	Studied blood vessel	Drug	Drug concentration	Effect on vasculature
Andriambeloson et al. (1998)	Male Wistar rats (12–14 w.o.; undisclosed animal weight)	Thoracic aorta	CA	10^–5^ – 3×10^–1^ g/L	Relaxation of intact and denuded vessels pre-contracted with norepinephrine
Taubert et al. (2002)	Female and castrated male pigs (7–9 m.o.; undisclosed animal weight)	Right coronary artery	CA	Concentration range not disclosed	Relaxation of intact vessels pre-contracted with PGF2_α_, abolished by endothelium removal and by L-NMMA (from pD_2_ values published in this paper, a concentration of 0.01 μM is inferred to have been used)
Cicala et al. (2003)	Male Wistar rats (200–250 g; undisclosed age)	Thoracic aorta	CAPE	0.1–300 μM	Concentration-dependent inhibition of phenylephrine-induced contraction
					Inhibition of KCl-induced contraction at high concentration (inhibition of intracellular calcium increase)
Long et al. (2009)	Pig (undisclosed animal age and weight)	Left anterior descending coronary artery	CAPE	CAPE 1–1000 μM	Relaxation of vessels pre-contracted with KCl or PGF2_α_. Relaxation was abolished by propranolol, methylene blue, L-NNA and SQ22536, but not by indomethacine
Çeçen et al. (2016)	Female Wistar-Albino rats (250–300 g; undisclosed age)	Thoracic aorta	CAPE	10, 100, 300 μM	Concentration-dependent inhibition of phenylephrine-induced contraction
Duman et al. (2014)	Human subjects (undisclosed data)	Umbilical arteries	CAPE	0.1–1000 μM	Relaxation of arteries pre-constricted with endothelin and PGF2_α_
Ho et al. (2013)	Healthy and streptozotocin-induced diabetic male Wistar rats (8 w.o.; 250–300 g)	Coronary arteries	CAPA	1, 3, and 10 μM administered intravenously to isolated hearts	Concentration-dependent increase in coronary blood flow in healthy and diabetic rats, although less pronounced in the latter
		Thoracic aorta		3 mg/kg CAPA administered intraperitoneally twice daily for 4 weeks	Concentration-dependent relaxation of endothelium-intact and endothelium-denuded aortae pre-constricted with phenylephrine.
					A right shift in the dose-response of phenylephrine-induced contraction. The effect was more pronounced in intact vessels.

#### Caffeic Acid

Caffeic acid shows moderate vasorelaxant activity. In PGF2_α_-precontracted porcine coronary arteries, CA induces the release of nitric oxide (NO) and vessel relaxation, most of which is abolished in denuded vessels or in intact vessels blocked with N^G^-monomethyl-L-arginine (L-NMMA, nitric oxide synthase (NOS) blocker), which suggests the importance of the endothelial NO-cyclic guanosine monophosphate (cGMP) pathway (Taubert et al., 2002). Conversely, in rat thoracic aortae pre-contracted with NE, there is no difference in the CA-induced relaxation between intact and denuded vessels, which indicates that this effect is endothelium-independent in this vessel (Andriambeloson et al., 1998). Therefore, the vasorelaxant activity of CA seems site-specific.

#### Caffeic Acid Phenethyl Ester

Of all the CA derivatives, CAPE is the single most widely studied compound for its vasorelaxant action. Studies on rat thoracic aortae have established that CAPE induces vasorelaxation through both an endothelium-dependent mechanism, which occurs with low concentrations, and an endothelium-independent mechanism occurring with high concentrations (Cicala et al., 2003; Çeçen et al., 2016).

##### Endothelium-dependent mechanism

Caffeic acid phenethyl ester causes the concentration-dependent relaxation of different types of intact vessels pre-contracted with PE (Cicala et al., 2003), ET (Duman et al., 2014), or PGF2_α_ (Duman et al., 2014), but not with KCl (Cicala et al., 2003; Long et al., 2009). Addition of N^G^-nitroarginine methyl ester (L-NAME, NOS inhibitor) to intact rat thoracic aortae pre-contracted with PE abolishes the vasorelaxant effect of CAPE at low concentrations (0.1–100 μM), with the same response being observed in endothelium denuded vessels (Cicala et al., 2003). Similarly, addition of L-NAME to human umbilical arteries pre-contracted with PGF2_α_ or ET also inhibits vasorelaxation (Duman et al., 2014). In porcine coronary arteries, vasorelaxation is partially abolished by N^ω^-nitro-L-arginine (L-NNA, NO synthase (NOS) inhibitor) and methylene blue (soluble guanyl cyclase inhibitor) (Long et al., 2009). These results strongly suggest that CAPE-mediated vasodilation is due to an increase in the endothelial NO-cGMP pathway, probably by enhancing the activity of endothelial NOS (eNOS). Furthermore, in human umbilical vein endothelial cells (HUVECs), CAPE (1–100 μM) increases intracellular NO content, an effect that is concentration-dependent. However, as the highest studied concentration (100 μM) actually decreases NO content with time, this suggests that impairment in endothelial cell viability may occur with CAPE at high concentrations (Kamil Burgazli et al., 2013). Furthermore, the endothelial secretion of vasodilator prostaglandins does not seem to be involved in CAPE-mediated vasorelaxation as indomethacin [cyclooxygenase (COX) inhibitor] does not affect the relaxation of porcine coronary arteries (Long et al., 2009).

##### Endothelium-independent mechanism – role of calcium channels

In both intact and denuded KCl-precontracted rat thoracic aortae, the vasorelaxant effect of CAPE is evident only at the highest studied concentration (100 μM), which suggests the existence of an endothelium-independent relaxation mechanism (Cicala et al., 2003). In fact, at high concentrations, CAPE also inhibits PE-triggered contraction in a calcium-free medium, suggesting that it prevents either the mobilization of calcium from intracellular stores or its influx across the VSM cell membrane (Cicala et al., 2003; Çeçen et al., 2016). In porcine coronary arteries, CAPE inhibits both KCl concentration-dependent contraction, but also reduces the calcium concentration-dependent contraction in a potassium-rich medium (Long et al., 2009). These results reveal CAPE’s calcium-antagonistic property and suggest that CAPE could inhibit calcium influx through voltage-gated channels in VSM cells.

##### Endothelium-independent mechanism – role of potassium channels

When propranolol (beta adrenergic receptor blocker) and N-nitro-L-arginine (L-NNA) are applied together to porcine coronary arteries, CAPE is still able to induce relaxation, which suggests the existence of yet another vasorelaxant mechanism. A possibility that is currently being considered is that CAPE acts on VSM potassium channels as their activation will lead to hyperpolarization and, consequently, to diminished activity of voltage-gated L-type calcium channels and vasorelaxation. This hypothesis has been proposed as CAPE is already known to stimulate the large conductance calcium-activated potassium channels (BK_Ca_) in rat pituitary GH3 cells (Lin et al., 2004) and human oral keratinocytes (Shieh et al., 2005). Although the potential of CAPE to modulate vascular BK_Ca_ channels still needs to be explored, there is evidence to suggest that it effectively contributes to the modulation of potassium channels in endothelial cells. Stimulating HUVECs with CAPE (1–100 μM) leads to a concentration-dependent increase in the cytosolic calcium concentration and, consequently, to hyperpolarization evoked by calcium-activated potassium channels. However, the latter has been observed only for the 10 and 100 μM concentrations (Kamil Burgazli et al., 2013). A rise in cytosolic calcium would lead to NO secretion which, downstream in its signaling pathway, would lead to VSM cell membrane hyperpolarization and to diminished calcium influx.

##### Role of beta receptors in vasorelaxation

Another mechanism for the vasorelaxant effect of CAPE seems to involve vascular beta adrenergic receptors. Alpha and beta adrenergic receptors are present in both endothelial and VSM cells (Sorriento et al., 2011). Stimulation of beta adrenergic receptors results in vasorelaxation through the activation of adenylate cyclase in VSM cells (Kuriyama et al., 1982) and of NOS in endothelial cells. There are opposing results about the role of adenylate cyclase in the vasorelaxation by CAPE. In intact porcine coronary arteries, propranolol (beta blocker) and SQ22536 (adenylate cyclase inhibitor) reduce CAPE-induced vasorelaxation, which suggests that the activation of beta receptors and cyclic adenosine monophosphate (cAMP) may be involved in this responde (Long et al., 2009). This assertion is logical given the partial structural homology that CAPE shares with the catechol ring of catecholamines, which could justify its action on adrenergic receptors (Cicala et al., 2003; Long et al., 2009). In contrast, in denuded rat aortae, CAPE-mediated relaxation is not affected by inhibition of adenylate cyclase (Cicala et al., 2003). This would suggest that CAPE’s action on beta receptors is more pronounced in endothelial cells rather than VSM cells, or CAPE’s action on endothelial cells might inhibit VSM cell contraction. This latter hypothesis is reasonable as the increase in intracellular cGMP (evoked by NO) and cAMP (evoked by beta receptor stimulation) inhibits calcium channels in VSM cell membranes (Ishikawa et al., 1993; Kelly et al., 2007).

#### Caffeic Acid Phenethyl Amide

Caffeic acid phenethyl amide also exhibits vasorelaxant action. In rat coronary arteries from isolated hearts, CAPA (1–10 μM) increases blood flow, which is partially abolished with L-NAME. This suggests that CAPA activates the endothelial NO-cGMP pathway (Ho et al., 2013), which has been attributed to CAPA’s radical scavenging activity that prevents NO degradation by reactive oxygen species (ROS). Although there are no studies that have yet tested this hypothesis, it has been proposed that CAPA can also increase manganese superoxide dismutase activity in the vasculature (Ho et al., 2013), similarly to its known effect on adipose tissue (Weng et al., 2012). Conversely, in intact and denuded rat thoracic aortae, CAPA (1, 3, 10, 30, 100, and 300 μM) shows concentration-dependent inhibition of potassium or PE-mediated constriction. In intact vessels this relaxation is not inhibited by L-NAME (NOS blocker), ODQ (NO-sensitive guanylate cyclase blocker) or methylene blue (soluble guanylate cyclase blocker). Endothelium removal exerts no difference in CAPA-mediated relaxation in PE-constricted vessels, but decreases the relaxation of potassium-constricted vessels. The same study has shown that CAPA also acts as a weak alpha-adrenergic receptor blocker (Ho et al., 2013). Taken together, these results apparently indicate that the molecular target of PE (alpha adrenergic receptor) is present in endothelial and VSM cells, whereas the target of potassium-induced contraction (voltage-gated calcium channel) seems more prevalent in VSM cells. Given the structural analogy to CAPE, in addition to its alpha adrenergic blocker activity, CAPA may also act as a potassium channel activator.

There is a notable shortage of studies that focus on exploring the effects of CA and its derivatives on perfusion *in vivo*. The few articles to have assessed perfusion *in vivo* have compared CAPE pre-treated and untreated animals subjected to ischemia-reperfusion injuries by using laser Doppler flowmetry as a technique to confirm ischemia during surgery (Zhou et al., 2006; Khan et al., 2007). To the authors’ knowledge, there are no ongoing clinical trials that employ CA or its derivatives for therapeutic intentions with cardiovascular diseases.

### Inhibitory Activity on the Renin-Angiotensin-Aldosterone Axis

Caffeic acid and several derivatives are known to modulate the renin-angiotensin-aldosterone endocrine axis *in vitro* (Bhullar et al., 2014). Caffeic acid phenethyl ester shows a higher renin inhibitory activity over CA, whereas CAPA shows weak activity. The derivative with the highest renin inhibition was a caffeic acid diphenylethyl ester [2,2-Diphenylethyl (2E)-3-(3,4-dihydroxyphenyl)acrylate], followed by a caffeic acid naphthylethyl ester [2-(2-Naphthyl)ethyl 3,4-dihydroxycinnamate]. Regarding the ability to inhibit angiotensin converting enzyme (ACE), CA, CAPE and the phenylpropyl ester of caffeic acid [3-Phenylpropyl (2E)-3-(3,4-dihydroxyphenyl)acrylate] show a strong activity. Caffeic acid phenethyl ester also shows an inhibitory activity and higher than CAPA. Most of these compounds showed weak inhibition activity against aldosterone. Caffeic acid phenethyl ester was the weakest compound, while CA, CAPA and the CA derivative propyl caffeate showed higher activities. Although these results derive from an *in vitro* study, they have been demonstrated recently in cyclosporine-induced hypertensive Sprague-Dawley rats, where CA significantly lowered plasma ACE activity (Agunloye et al., 2019). Further research is needed to assess whether other CA derivatives share these activities *in vivo* and whether they contribute significantly to blood pressure regulation.

### Effect on Heart Rate and Blood Pressure

To the authors’ knowledge, to the present date there are no published studies exploring the effect of administering CA or its derivatives on cardiovascular variables (i.e., blood pressure, heart rate) in humans. The only available studies have assessed the impact of consuming hydroxycinnamic-rich foods on biochemical and physiological variables, including blood pressure. Although most studies suggest that a high dietary intake of hydroxycinnamic-rich foods confers protection against cardiovascular disease, especially in high risk patients, it cannot be inferred that this benefit is attributed solely to CA, CAPE or any other derivative (Martini et al., 2019). The effects of CA and its derivatives on cardiovascular variables assessed *in vivo* in preclinical studies are detailed in Table 2.

**TABLE 2:** Description and main results of the studies (*in vivo*, *ex vivo*) characterizing the effect of caffeic acid and its derivatives on cardiovascular physiological variables (CA–caffeic acid; CAPE–caffeic acid phenethyl ester; CAPA–caffeic acid phenethyl amide; w.o.–weeks old; CAO–coronary artery occlusion; MCAO–middle cerebral artery occlusion).

Authors	Species/strain (sample size, age, weight), experimental procedure and anesthetic scheme	Drug	Drug dose and administration route	Effect on cardiovascular physiological variables
Leeya et al. (2010)	Healthy adult female Wistar rats (*N* = 6, 210–250 g) under pentobarbital anesthesia	CA	1–10 mg/kg administered intravenously	Mean blood pressure and heart rate decreased only at a 10 mg/kg dose
Zhou et al. (2006)	Adult male Wistar rats (*N* = 6, 210–250 g) subjected to middle cerebral artery occlusion (MCAO) under chloral hydrate anesthesia	CA	10 and 50 mg/kg administered intraperitoneally 30 min before MCAO, 0,1 and 2 h after reperfusion on the first day, then twice daily from days 2 to 5	No difference in blood pressure and cerebral blood flow among sham, ischemic and ischemic + CA groups
Yokozawa et al. (1995)	Male Wistar rats (*N* = 6; 200–210 g) with adenine-induced renal failure and consequent hypertension (no anesthesia)	CA	10 mg/kg administered orally in drinking water for 24 days	No difference in systolic and mean blood pressure
Agunloye et al. (2019)	Cyclosporine-induced hypertensive rats (*N* = 6, undisclosed strain, gender, age and weight; no anesthesia)	CA	10 mg/kg/day and 15 mg/kg/day orally administrated for 7 days	Significant decrease in systolic blood pressure and heart rate
Iraz et al. (2005a)	Healthy male Sprague-Dawley rats (*N* = 6; 12 w.o.; 250–300 g) under urethane anesthesia	CAPE	1, 5, 10, and 20 mg/kg administered intravenously	Dose-dependent decrease in mean blood pressure and heart rate; 1 mg/kg lowered the mean blood pressure up to 20 s and 5 and 10 mg/kg up to 2 min; 10 mg/kg decreased heart rate up to 10 min; 20 mg/kg caused death after a few seconds
Iraz et al. (2005b)	Healthy (*N* = 8) and bivagotomized (*N* = 8) male Sprague Dawley rats (12 w.o.; 250–300 g) under urethane anesthesia	CAPE	5 mg/kg administered intravenously	The mean blood pressure was lowered up to 1 min
Hassan et al. (2014)	Healthy, insulin-resistant and insulin-resistant male Wistar rats (*N = 8*; 85–155 g; undisclosed age) without anesthesia	CAPE	30 mg/kg/day administered orally by gavage for 6 weeks	In healthy animals, systolic, diastolic and pulse blood pressure did not change
				In insulin-resistant and insulin-deficient animals, CAPE alleviated the increase in systolic, diastolic and pulse blood pressure
Gun et al. (2016)	High fructose consumer Sprague Dawley rats (8 w.o.; undisclosed gender and weight) without anesthesia	CAPE	50 μmol/kg/day administered intraperitoneally for 2 weeks	CAPE significantly ameliorated the increase in blood pressure that accompanied vascular damage
Eşrefoglu et al. (2005)	Male Wistar rats (*N = 8*; 250–350 g; undisclosed age) subjected to CAO under urethane anesthesia	CAPE	50 μmol/kg administered intravenously 10 min before and during occlusion	CAPE prevented the drop in blood pressure induced by I/R injury and accelerated recovery to pre-injury values
Cagli et al. (2005)	Male Wistar rats (250–350 g, undisclosed age and sample size) subjected to CAO under thiopental anesthesia	CAPE	10 μmol/kg administered intravenously 10 min before occlusion	No differences in heart rate between the control and CAPE-treated groups
Ozer et al. (2004)	Male Wistar rats (*N* = 7; 250–350 g) subjected to CAO under urethane anesthesia	CAPE	1.25 μM/kg/min administered intravenously 10 min before and during occlusion by infusion	No changes in blood pressure and heart rate between the control and CAPE-treated animals
Parlakpinar et al. (2005)	Male Wistar rats (*N* = 8; 10–12 w.o.; 200–250 g) subjected to CAO under urethane anesthesia	CAPE	50 μmol/kg administered intravenously before and during occlusion	No difference in heart rate or blood pressure in the control, ischemic or CAPE-treated groups. CAPE exerted no effect during ischemia or reperfusion
Altuǧ et al. (2008)	New Zealand white male rabbits (*N* = 6; 2.5–3 kg) subjected to MCAO under ketamine, xylazine and atropine anesthesia	CAPE	10 μmol/kg/day intraperitoneally after MCAO for 7 days	No differences in blood pressure in CAPE-treated and control groups
Khan et al. (2007)	Male Sprague–Dawley rats (240–260 g; undisclosed age and sample size) subjected to MCAO under xylazine-ketamine anesthesia	CAPE	1,2,5, and 10 mg/kg administered intravenously during MCAO or after reperfusion	No difference in blood pressure and heart rate between the control and animals treated with CAPE during cerebral I/R injury
				Cerebral blood flow increased significantly at a dose of 2 mg/kg in comparison to controls
Tsai et al. (2006)	Male Long–Evans rats (270–350 g) subjected to MCAO under halothane anesthesia	CAPE	0.01, 0.1, 1, or 10 μg/kg administered intravenously 15 min before MCAO	No significant changes in heart rate or blood pressure between groups
Fadillioglu et al. (2004)	Male Sprague-Dawley rats (60 days old) receiving doxorubicin under urethane anesthesia	CAPE	10 μmol/kg/day administered intraperitoneally for 12 days	CAPE attenuated the doxorubicin-induced increase in heart rate and blood pressure
Mollaoglu et al. (2006)	Adult males Wistar Albino rats (*N* = 12; 8 w.o., 280 g) receiving cadmium chloride i.p., for 15 days (undisclosed use of anesthesia)	CAPE	10 μmol/kg/day administered intraperitoneally for 15 days	No significant changes in heart rate or blood pressure
Castellano et al. (2016)	Male Sprague Dawley rats (275–325 g; undisclosed age and sample size)	CAPE	40 μM	In heart preparations CAPE restored L-NAME-induced compromise in left ventricle diastolic and end-systolic pressures after I/R injury
Chang et al. (2013)	Adult male Hartley guinea-pigs (300–350 g, *N* = 9–10; undisclosed age)	CAPE	1, 3, and 10 μM for cardiac electrical conduction studies	Negative chronotropic effect and frequency-dependent depression of AV nodal conduction.
				Reduction in the occurrence of reperfusion-induced ventricular fibrillation
			3, 10, 30, 100 μM for cardiac contraction studies	Decrease in left ventricular pressure
Ho et al. (2013)	Healthy (*N* = 5) and diabetic (*N* = 9) male Wistar rats (8 w.o.; 250–300 g), under sodium pentobarbital anesthesia	CAPA	1, 3, and 10 μM intravenously in heart preparations	No change in heart rate
		CAPA	1, 5, and 10 mg/kg administered orally by gavage	No changes in blood pressure (data not shown in paper)

#### Caffeic Acid

Caffeic acid administered intravenously at 10 mg/kg to healthy female Wistar rats under pentobarbital anesthesia lowers blood pressure and heart rate (Leeya et al., 2010). However, doses of 10 or 50 mg/kg delivered intraperitoneally are unable to lower blood pressure in rats undergoing cerebral artery occlusion under chloral hydrate anesthesia (Zhou et al., 2006). Given CA’s lipophilic character, it is improbable that this difference in response should be attributed to the administration route. It could be rather attributed to differences in vascular and/or autonomic reactivity to the employed anesthetic schemes. However, a similar lack of hypotensive effect is observed when CA is administered orally to conscious rats with adenine-induced renal failure and cyclosporine-induced hypertension. When administered orally at a 10 mg/kg/day dose for 24 days to rats with renal failure (and hypertension), CA fails do decrease blood pressure (Yokozawa et al., 1995). When administered orally at doses of 10 and 15 mg/kg/day for 7 days to cyclosporine-induced hypertensive rats, CA decreases systolic blood pressure and heart rate. In this study, plasma ACE and heart acetylcholinesterase and butyrylcholinesterase activities were decreased, as well as plasma and heart arginase activities (Agunloye et al., 2019). From these results, it can be inferred that CA increased the effect of acetylcholine on the heart and decreased plasma angiotensin II levels, thus probably having decreased blood pressure through modulation of the cardiac output and peripheral vascular resistance. Therefore, when considering the oral administration route, present data suggests that treatment duration is a major determinant of CA’s hypotensive effect. Nevertheless, it is also probable that the oxidative stress underlying the pathophysiology in these animal models might oppose CA’s hypotensive effect.

#### Caffeic Acid Phenethyl Ester

Caffeic acid phenethyl ester administered intravenously to Sprague-Dawley rats lowers heart rate and mean blood pressure levels in a dose-dependent manner (Iraz et al., 2005a). A dose of 1 mg/kg lowers blood pressure for up to 20 s, whereas the effect lasts up to 2 min at 5 and 10 mg/kg. A dose of 20 mg/kg causes death within seconds, probably due to hemodynamic shock caused by severe hypotension and bradycardia (Iraz et al., 2005a). In rats subjected to bilateral vagotomy or receiving atropine, the negative chronotrope effect of CAPE at a dose of 5 mg/kg is suppressed, suggesting that CAPE stimulates the parasympathetic nervous system on the heart (Iraz et al., 2005b).

Caffeic acid phenethyl ester has shown the ability to stimulate beta adrenergic receptors in VSM cells as well as to potentiate the release of NO from the vascular endothelium. Given that there are no studies investigating whether CAPE exerts similar actions on cardiomyocytes, current evidence suggests that it solely modulates cardiac parasympathetic activity. Given that neither vagotomy nor atropine prevent CAPE’s hypotensive effect (Iraz et al., 2005b), the latter must be attributed to its vasorelaxant effect. It can, thus, be assumed that CAPE-mediated vasorelaxation is potent enough, together with its bradycardic effect, to lower blood pressure. These studies report a minor difference in the duration of the blood pressure lowering effects of CAPE. In the first study (Iraz et al., 2005a), CAPE lowered blood pressure for up to 2 min, which went down to 1 min in the second study (Iraz et al., 2005b). As equal experimental conditions were used in both studies, this difference in effect could be attributed to the difference in sample size at least.

Oral CAPE administration seems to lose potency versus intravenous and intraperitoneal administrations, at least in healthy animals. A dose of 30 mg/kg/day administered by oral gavage for 6 weeks did not significantly change the blood pressure of healthy Wistar rats (Hassan et al., 2014). As an intravenous dose of 20 mg/kg was certain to lower both heart rate and blood pressure of healthy Sprague-Dawley rats (Iraz et al., 2005a), the lack of a measurable response in this case should be primarily attributed to the administration route, although strain-specific pharmacokinetics may also be considered. Under these conditions, the intestinal and hepatic first pass effects are apparently responsible for diminishing CAPE bioavailability and, consequently, the dose that reaches the bloodstream. Still, insulin-resistant and insulin-deficient rats benefit from oral CAPE administration (30 mg/kg/day) as it ameliorates the rise in blood pressure, an effect attributed to CAPE-mediated improvement of vascular reactivity and stiffness (Hassan et al., 2014). This beneficial effect was not observed in healthy controls, suggesting that orally administered CAPE may exert a higher impact in animals with a higher vascular tone, a hallmark of insulin resistance and insulin deficiency (El-Bassossy et al., 2012). In high fructose consumer diabetic rats, subacute CAPE administrations (50 μmol/kg/day intraperitoneally for 2 weeks) ameliorate the rise in blood pressure that accompanies this metabolically mediated vascular damage, and was attributed to increased eNOS levels, even though an anti-inflammatory effect cannot be ruled out (Gun et al., 2016). Furthermore, as CAPE is also able to reduce insulin resistance (Hassan et al., 2014), it can be argued that this blood pressure-lowering effect might also be attributed to increased vasculature sensitivity to insulin’s vasorelaxant action.

Blood pressure has been monitored in several studies of ischemia/reperfusion (I/R) injuries, and CAPE’s vasorelaxant activity has been assessed. In the vast majority of these studies, CAPE failed to lower blood pressure in comparison to control groups. Given that different experimental conditions were involved, including administration routes, CAPE doses, and anesthetic schemes, these factors seem to be the primary causes for the apparent lack of blood pressure response. In studies into coronary artery I/R, at first glance the CAPE dose would seem to explain lack of response. When low doses are used (1–10 μM/kg), no effect is observed (Cagli et al., 2005; Ozer et al., 2005b; Parlakpinar et al., 2005), whereas with a higher dose (50 μM/kg) an amelioration of increased blood pressure takes place (Eşrefoglu et al., 2005). Surprisingly, in a different study with practically identical experimental conditions, the same high dose failed to produce a beneficial effect on blood pressure (Parlakpinar et al., 2005), which might justify the need to improve this experimental model in terms of reproducibility. At lower doses, it can also be argued that I/R-mediated oxidative stress suffices to offset CAPE’s vasorelaxant and blood pressure-lowering action. This has, once again, been observed in middle cerebral artery I/R studies, where low concentrations failed to have a beneficial effect on blood pressure (Tsai et al., 2006; Khan et al., 2007; Altuǧ et al., 2008).

Blood pressure and heart rate have also been monitored in toxicity studies. In Sprague-Dawley rats, 10 μmol/kg/day for a 12-day pretreatment with CAPE administered intraperitoneally attenuated the doxorubicin-mediated increase in heart rate and blood pressure (Fadillioglu et al., 2004). In contrast, the same dose administered intraperitoneally for 15 days to Wistar rats did not significantly ameliorate the cadmium-induced increase in blood pressure despite its beneficial effects on myocardial and vascular histology (Mollaoglu et al., 2006). In this case, the differences in animal strain, treatment duration and general mechanism of lesion can be attributed to these seemingly opposing results. Finally, it has been proposed that CAPE might not produce significant changes in blood pressure due to opposing actions on primary blood pressure determinants. On the one hand, CAPE may have a positive inotropic agent (induction of beta adrenergic receptor by NO) and promote coronary vasodilation by increasing cardiac output; on the other hand it may decrease peripheral resistance by its vasorelaxant effect. In this way, the mean blood pressure might remain stable (Ilhan et al., 2014). For this reason, the effect of CAPE and other CA derivatives on hemodynamics should be clarified *in vivo* by simultaneously assessing perfusion and blood pressure.

Caffeic acid phenethyl ester has also demonstrated an antiarrhythmic effect on an animal model of myocardial infarction, which has not attributed to direct action in cardiomyocytes, but to indirect actions via ROS scavenging, and possibly vasorelaxant effects (Huang et al., 2005). In isolated guinea pig hearts, CAPE displays a negative chronotropic effect and frequency-dependent depression of atrioventricular nodal conduction. CAPE also exhibits an antiarrhythmic effect by reducing the occurrence of reperfusion-induced ventricular fibrillation and decreased left ventricular pressure. These effects are not attributed to CAPE’s antioxidant or anti-inflammatory effects, rather to its ability to interact with cardiomyocyte ion channels. Although CAPE also acts on potassium channels (slight inhibition of delayed outward channels, with no effect on inward rectifier channels), these effects are attributed to the inhibition of inward sodium and calcium currents (Chang et al., 2013).

#### Caffeic Acid Phenethyl Amide

Caffeic acid phenethyl amide administered orally by gavage (1, 5, and 10 mg/kg) does not change blood pressure in Wistar rats, but a weak alpha-adrenergic receptor blocking effect is recognized. When administered to heart preparations, concentrations of 1, 3, and 10 μM do not significantly lower heart rate, which suggests that, as with CAPE, the central nervous system may be required for CAPA’s possible bradycardic effect (Ho et al., 2013).

### Anti-inflammatory and Immunomodulatory

Caffeic acid phenethyl ester is able to inhibit COX (Jung et al., 2008) and lipoxygenase (LOX) (Doiron et al., 2017) enzymes. It is also thought to inhibit the release of arachidonic acid from the cell membrane and, consequently, both the activity of COX-1 and -2 are suppressed. Furthermore, CAPE has been also shown to suppress COX-2 gene expression (Chung et al., 2004; Jung et al., 2008). In contrast, CA is known to inhibit 5- and 12-LOX activity (Yasuko et al., 1984; Doiron et al., 2017), but not COX (Yasuko et al., 1984). Other derivatives have also displayed important inhibiting properties on macrophage NO secretion, which reduces oxidative stress (Zhang J. et al., 2014). Several other CA and CAPE derivatives have been synthesized in recent years to enhance their anti-inflammatory action. For example, the substitution of hydroxyl groups in CAPE’s catechol ring by methoxy groups is reported to increase its anti-inflammatory action (Zhang P. et al., 2014). Additionally, CAPE is an immunomodulatory molecule which suppresses the transcription of nuclear factor κB (NF-κB) to lower the expression levels of inflammatory cytokines, such as interleukins 1, 6 and tumor necrosis factor alpha (TNF-α) (Natarajan et al., 1996). It is believed that CAPE suppresses NF-κB activation by inhibiting its interaction with DNA (Song et al., 2002), and by inhibiting the degradation of inhibitor of nuclear factor kappa B alpha (IκBα) (Li et al., 2000). In fact, it has been suggested that the anti-inflammatory effect of CAPE is most likely due to ROS inhibition at the transcriptional level through suppression of NF-κB activation and the direct inhibition of NOS activity (Ito et al., 2005). Finally, CAPE’s anti-inflammatory and immunomodulatory effects are also thought to be mediated by the reduction of c-jun-N-terminal kinase activity and the regulation of mitogen activated protein kinases (MAPK) signaling pathways (Wang L.C. et al., 2009).

### Antioxidant and Radical Scavenging Effects

The roles of reactive oxygen and nitrogen species (ROS and RNS, respectively) in physiological and pathophysiological processes have become increasingly clear in the past decades. ROS constitute various forms of activated oxygen, including free radicals such as superoxide anion radicals $\left({\text{O}}_{2}^{\cdot -}\right),$ hydroxyl radicals (OH^⋅^) and non-free radical species such as hydrogen peroxide (H_2_O_2_) and singlet oxygen (O21). Reactive oxygen species (ROS) are produced by normal physiological processes and play important roles in tissue homeostasis and cell signaling pathways (Carolina et al., 2018). Reactive oxygen species are produced by several mammalian cell enzymes, such as electron transport chain complexes I and III, nicotinamide adenine dinucleotide phosphate (NADPH)-oxidase (NOX), xanthine oxidoreductase (XOR), myeloperoxidase (MPO), NOS and cytochrome P450 enzymes. Nicotinamide adenine dinucleotide phosphate oxidase enzymes catalyze the reduction of molecular oxygen to superoxide anion using NADPH as an electron donor. The sole function of NOX is ROS production for the oxidative burst in phagocytes to kill bacteria. Superoxide has a very short half-life and is rapidly converted to the less-reactive hydrogen peroxide by superoxide dismutase (SOD) enzymes, with hydrogen peroxide being continuously decomposed into water and oxygen by catalase (CAT) enzymes (Burgoyne et al., 2012). Xanthine oxidoreductase is involved in the oxidation of hypoxanthine to xanthine and furthermore to uric acid. It is naturally expressed in the dehydrogenase (XDH) form but, under inflammatory conditions, can be converted into the oxidase (XO) form, which generates superoxide and hydrogen peroxide radicals from molecular oxygen. Myeloperoxidase, found in neutrophils, catalyzes the oxidation of chloride by hydrogen peroxide to form the strong oxidant hypochlorous acid (HOCl), the prime mediator of oxidative killing in the phagosome of phagocytes. Finally, NOS catalyzes the conversion of L-arginine to nitric oxide (NO) and citrulline using molecular oxygen and reduced nicotinamide-adenine-dinucleotide phosphate (NADPH) as co-substrates. The activity of NOS is indirectly modulated by the enzyme arginase, which catalyzes the conversion of L-arginine to ornithine (Durante et al., 2007). Nitric oxide is a local mediator with a vast importance in cardiovascular homeostasis. It acts as a potent vasorelaxant, inhibits platelet aggregation and adhesion, inhibits the interaction of leukocytes with endothelial cells as well as the proliferation and migration of VSM cells (Huang et al., 1995; Loscalzo and Welch, 1995; Furchgott, 1996). Because of these effects, NO exerts an important anti-atherosclerotic activity (Kawashima and Yokoyama, 2004). Nitric oxide can also react with superoxide to generate peroxynitrite (OONO^–^), the representative of the reactive nitrogen species (RNS), to also damage cells (Weidinger and Kozlov, 2015). Normally, ROS and RNS are removed by cellular antioxidant systems, such as GSH, glutathione peroxidase (GSH-Px), SOD and CAT. However, when aberrant ROS and RNS production exceeds cells’ antioxidant buffering capacity, oxidative and nitrosative stress ensues, respectively, to damage host biological structures like proteins, DNA, and lipids, which can promote inflammation and programmed cell death (Verma et al., 2002; Yellon and Hausenloy, 2007). Cellular or organelle membranes, owing to their high polyunsaturated fatty acids, are particularly susceptible to ROS damage. This type of lipid damage, termed lipid peroxidation, is a process in which free radical species remove electrons from lipids and subsequently produce reactive intermediates that can undergo further reactions. Damaged phospholipids disrupt cellular functions and can also act as cell death signals, inducing programmed cell death (Que et al., 2018). During oxidative and/or nitrosative stress, cellular antioxidant systems (i.e., GSH) are wasted and oxidative products (i.e., malonaldehyde, MDA) increase. In general, the oxidative status of the organism affects many organic functions, including cardiovascular and metabolic. Oxidative and nitrosative stress play important roles in the pathophysiology of cardiovascular diseases, affecting disease progression and clinical outcomes (D’Oria et al., 2020).

#### Activity on NOS Isoenzymes

Nitric oxide synthase comprises the neural (nNOS), inducible (iNOS), and endothelial (eNOS) isoenzymes, which differ in terms of the magnitude and duration by which they release NO. Endothelial and neural NOS are constitutively expressed and release low quantities of NO over long periods of time, exerting several protective effects. Endothelial NOS dilates all types of blood vessels by stimulating soluble guanylyl cyclase and increasing cGMP in VSM cells, where its absence leads to hypertension. When released toward the vascular lumen NO inhibits platelet aggregation and adhesion to the vascular wall, prevents release of platelet-derived growth factors that stimulate smooth muscle proliferation and its production of matrix molecules. Furthermore, it also inhibits leucocyte adhesion and vascular inflammation (Förstermann and Sessa, 2012). Caffeic acid increases the endothelial production of NO in HUVECs by inducing the expression and increasing the activity of eNOS (Wallerath et al., 2005; Gun et al., 2016) or by scavenging ROS, which prevents NO destruction (Migliori et al., 2015). *In vivo* studies seem to support that CA and CAPE enhance eNOS expression (Khanna et al., 2005; Long et al., 2009; Gun et al., 2016).

Inducible NOS is usually not constitutively expressed, only being upregulated in macrophages after exposure to certain molecular triggers such as endotoxins, cytokines and lipid mediators (Laroux et al., 1995). Macrophages then release large quantities of NO in short periods of time which exert cytotoxic effects on parasitic microorganisms and tumor cells by inhibition in enzymes involved in cellular energetics and DNA stability and replication. Besides being a hallmark of the large majority of auto-immune and inflammatory and diseases, including neurodegeneration, abundant iNOS-generated NO plays a crucial role in septic shock, especially in microvascular damage and life-threatening hypotension (Förstermann and Sessa, 2012). Caffeic acid phenethyl ester is known to inhibit iNOS expression via suppression of NF-κB pathway, as well as to directly interfere with its catalytic activity (Song et al., 2002). Both CA and CAPE ameliorate neurodegeneration in I/R and chemical injuries in the brain in part by inhibiting iNOS expression. Interestingly, in a study conducted in mice with diabetic nephropathy, where kidney NO content is decreased, CAPE and *para*-nitro-CAPE enhance the expression of iNOS, preventing further kidney damage (Wang X. et al., 2017). Therefore, it would appear that the activity of CA and its derivatives on iNOS depends on the redox status of the tissue.

Neuronal NOS is involved in several synaptic events including long-term potentiation and long-term inhibition, being implicated in learning, memory and neurogenesis, as well as in the central regulation of blood pressure (Förstermann and Sessa, 2012). However, nNOS overactivity following neuron damaging events (e.g., infarct, inflammation, and excitotoxicity) can greatly increase the concentration of NO in the brain, leading to oxidative stress and neurotoxicity (Malinski et al., 1993). In brain I/R injury, both iNOS and nNOS are known to be responsible for NO accumulation and consequent neurotoxicity, whereas eNOS shows a neuroprotective role (Bolaños and Almeida, 1999). Therefore, inhibition of nNOS activity would be expected to improve the pathophysiological outcome. However, the neuroprotection associated with CA and CAPE treatment is predominantly associated with the inhibition of iNOS and COX-2, rather than that of nNOS. In fact, in Sprague–Dawley rat pups with brain I/R injury the expression of iNOS is increased, whereas that of nNOS is not. Pretreatment with CAPE at a dose of 40 mg/kg i.p., for 7 days significantly prevents I/R-induced neonatal brain damage in the cortical, hippocampal and thalamic regions. It inhibits calcium-induced cytochrome c release as well as iNOS and caspase-1 expression, whereas it does not affect nNOS expression (Wei et al., 2004). Similarly, in a C57BL/6 mouse model of 1-methyl-4-phenyl-1,2,3,6-tetrahydropyridine (MPTP)-induced Parkinson’s disease, orally administered CAPE (2, 5, or 10 mg/kg/day) for 7 days attenuates dopaminergic degeneration via inhibition of iNOS and caspase-1 expression, whereas it does not affect nNOS expression (Fontanilla et al., 2011). Again, in MPTP-mediated brain inflammation in C57BL/6 mice, CA (0.5, 1, and 2 g per 100 g diet) administered either before or after I/R injury induction for 4 weeks shows neuroprotective effects by inhibiting the expression of COX-2 and iNOS. The expression of nNOS is decreased when CA is administered before but not after I/R injury (Tsai et al., 2011). In conclusion, further studies should be conducted to confirm the effect of CAPE on nNOS expression in different animal models.

#### Structure-Activity Relationship

The antioxidant activity of CA and its derivatives, at least in cellular systems *in vitro*, the closest to *in vivo* conditions, is dependent not only on the molecular structure, but also on the solubility, hydrophobicity and stability of the compounds (Wu W.M. et al., 2007). Caffeic acid and CAPE are able to scavenge both reactive oxygen and nitrogen species, in acellular and in cellular systems (Yan-Chun and Rong-Liang, 1991; Kono et al., 1997; Wu W.M. et al., 2007; Kassim et al., 2014). In the former CA and CAPE inhibit lipid peroxidation to a higher extent than α-tocopherol (Wu W.M. et al., 2007; Göçer and Gülçin, 2011). Caffeic acid prevents the chain initiation of lipid peroxidation by scavenging peroxyl radical (LOO^⋅^) (Wu W.M. et al., 2007). Caffeic acid has also been shown to maintain proteins against oxidation by scavenging ROS and by assisting in their repair through transfer of electrons to amino acid radicals (Zhu et al., 2006). Additionally, part of the antioxidant effectiveness of CA and CAPE may lie in their ability to chelate transition metals such as iron and copper (Nardini et al., 1995). Finally, by inhibiting transcription factors (i.e., NF-κB) and enzymes (i.e., COX and LOX) indirectly involved in oxidative stress, CA and CAPE also exert important antioxidant activity. Antioxidant and free radical scavenging activities are attributed to several functional groups in the CA molecular structure, with the *ortho*-dihydroxyl functionality in the catechol ring being probably the best studied so far (Son and Lewis, 2002; Wu W.M. et al., 2007; Wang X. et al., 2010; Doiron et al., 2017). When CA is oxidized by ROS its intermediate radicals can be stabilized by these adjacent electron-donating hydroxy functions, and the free radical chain reaction may be terminated (Graf, 1992). By containing an unsaturated bond the CA ethylenic side chain ensures greater hydrogen donating ability and is therefore able to stabilize the phenoxy radical by resonance or to provide an additional site for reaction with ROS (Graf, 1992; Barone et al., 2009; Mathew et al., 2015). In fact, this side chain is responsible for the relatively greater activity of hydroxycinnamic acids compared to their hydroxybenzoic acid counterparts (Göçer and Gülçin, 2011). The esterification of caffeic acid forms compounds with increased lipophilicity, which might facilitate their interaction with membrane phospholipids and enhance their antioxidant properties, especially against lipid peroxidation (Son and Lewis, 2002; Wu W.M. et al., 2007). With regards to CAPE and its derivatives, the ester function is thought to play an important role in NO inhibition (Nagaoka et al., 2003). Finally, the presence of hydrogen-donating substituents such as the –NH group can also be responsible for enhancing the antioxidant activity of CA amidated derivatives (Son and Lewis, 2002; Barbakadze et al., 2005).

#### Cytoprotection on Endothelial Cells

Essentially due to their radical scavenging and antioxidant properties, CA and its derivatives have also shown cytoprotective effects on cardiovascular cells *in vitro*, such as vascular endothelial cells and cardiomyocytes. Caffeic acid, CAPE and CAPA show cytoprotective effects in human umbilical vein endothelial cells (HUVECs). Caffeic acid enhances basal and acetylcholine-induced endothelial NO production in a dose-dependent manner under hypoxia and while incubated with uremic toxins. This is not attributed to the induction of eNOS by CA but rather to its ROS scavenging activity, by which it prevents oxidative stress (Migliori et al., 2015). In fact, CA prevents oxidation of low-density lipoprotein (LDL) and the consequent rise in calcium concentration, therefore preventing apoptosis (Vieira et al., 1998). Dihydrocaffeic acid (DHCA) prevents oxidative stress in EA.hy926 endothelial cells, a permanent cell line derived from HUVECs that expresses factor VIII antigen, oxidizes LDL, and displays calcium-dependent stimulation of eNOS. In this cell line DHCA scavenges ROS and increases eNOS expression (Huang et al., 2004). Caffeic acid phenethyl ester produces concentration-dependent protection against menadione-induced cytotoxicity in HUVECs, and also prevents apoptosis. However, at high doses CAPE’s cytoprotective effect decreases, probably due to cytotoxicity. Curiously, this cytoprotective effect is shared by two CAPE fluorinated analogs but not by CA itself (Wang X. et al., 2006), which should prompt further research into the structure-activity relations. Again in menadione-induced toxicity endothelial cells, CAPE’s cytoprotective effect is mediated by the upregulation of heme oxidase-1 (HO-1) via transcriptional activation (Wang X. et al., 2008). Heme oxygenase-1 catalyzes the conversion of heme group into biliverdin, carbon monoxide and iron. Biliverdin is then converted to bilirubin by bilirubin reductase. Reactive oxygen species react with bilirubin, converting it back to biliverdin. By increasing the pool of biliverdin, HO-1 acts as an antioxidant enzyme, shifting the redox state to a reduce state and decreases superoxide formation (Turkseven et al., 2005). Interestingly, in fluorinated CAPE derivatives, cytoprotective effect is correlated with the upregulation of HO-1 but not with direct antioxidant effect (Wang X. et al., 2010). Caffeic acid phenethyl amide and two fluorinated compounds (FCAPA-1, FCAPA-2) have also shown cytoprotective effects in HUVECs. In particular, these compounds were shown to protect HUVECs against hydrogen peroxide-induced oxidative damage (Wang T. et al., 2008; Yang J. et al., 2010). Other CAPE and CAPA fluorinated compounds were shown to be toxic, although the origin for these differences is presently not clear.

#### Cytoprotection on Cardiomyocytes

Caffeic acid, CAPE and CAEA display cardioprotective properties in several animal models of myocardial injury mediated by hydrogen peroxide (Izuta et al., 2009), cadmium (Mollaoglu et al., 2006), doxorubicin (Fadillioglu et al., 2004), rotenone (Akpinar et al., 2005), isoproterenol (Oktar et al., 2010; Paeng et al., 2015) and electromagnetic radiation (Ozguner et al., 2005; Mansour and Tawfik, 2012). Pyrrolidinyl caffeamide (PLCA) shows higher ROS scavenging and cytoprotective effects than CA in cardiomyocytes exposed to hydrogen peroxide (Ku et al., 2016). Caffeic acid protects against isoproterenol-induced myocardial infarction in Wistar rats. Isoproterenol toxicity leads to the decrease of several antioxidant enzymes in cardiomyocytes including SOD, CAT, and GSH-Px and to the increase of lipid peroxides. Pretreatment with CA (15 mg/kg for 10 days) increased SOD, CAT, and GSH-Px activities, decreased cardiac peroxidation markers and improved myocardial tissue histology (Kumaran and Prince, 2010). Caffeic acid ethanolamine (CAEA) has shown superior myocardial-protective activity compared to CA itself. In isoproterenol-induced cardiomyocyte toxicity, CAEA improves cellular and mitochondrial oxidative stress by increasing manganese SOD activity. Furthermore, it reverses cellular energetics and preserves cardiac mechanical performance in a sirtuin-dependent mechanism and prevents cardiac remodeling, namely ventricular hypertrophy fibrosis (Lee et al., 2015b). Caffeic acid phenethyl ester also displays cardiac remodeling properties. In a model of pressure overload-induced myocardial hypertrophy, CAPE attenuates cardiac remodeling through down-regulation of the MEK/ERK signaling pathway *in vivo* and *in vitro* (Ren et al., 2017). Long-term CAPE supplementation (15 mg/kg/day for 95 days i.p.) in Sprague-Dawley rats increases the levels of antioxidant mediators and lowers MDA levels in the cardiomyocytes and aorta of senescent animals, which suggests that long-term supplementation may prevent both myocardial damage and vascular remodeling (Eşrefoglu et al., 2011). The fibrosis preventing activity of CAPE may be associated with the inhibition of myofibroblast formation and collagen synthesis (Mia and Bank, 2016). In an animal model of cadmium-induced atrial and ventricular hypertrophy, CAPE’s cardioprotection seems to be attributed at least to its antioxidant activity, namely the inhibition of lipid peroxidation and reduction of MDA and NO (Mollaoglu et al., 2006). Caffeic acid phenethyl ester (CAPE) also protects rat cardiomyocytes from hydrogen peroxide-induced oxidative injury by lowering MDA levels and increasing SOD and GSH-Px activities (Izuta et al., 2009). Early treatment with CAPE (10 mM/kg i.p. for 7 days) also protects Sprague-Dawley rat hearts from gamma radiation-induced injury owing to its antioxidant activity, as shown by the down-regulation of MDA, XO and adenosine deaminase (ADA), together with the up-regulation of nitrate/nitrite and SOD activity (Mansour and Tawfik, 2012). In rats subjected to electromagnetic radiation (900 MHz, 30 min/day, for 10 days) to evoke ROS production, CAPE (10 mM/mL/kg/day i.p., for 10 days) reduces MDA levels in the myocardium, while increasing the activity of SOD, CAT and GSH-Px (Ozguner et al., 2005). In a rat model of endotoxin-induced cardiac stress, CAPE restores several parameters indicating cardiomyocyte injury, including creatine kinase (CK) and lactate dehydrogenase (LDH), MDA, TNF-α, GSH, serum and cardiac nitrite/nitrate, as well as GPx and MPO activities, besides inducing HO-1 levels (Motawi et al., 2011). Finally, by suppressing NF-κB pathway, CAPE decreases the expression of iNOS and COX-2 enzymes and the release of pro-inflammatory cytokines such as TNF-α, leading to the decrease of ROS production (Natarajan et al., 1996).

### Role in Ischemia/Reperfusion Injury

Ischemia is an important trigger for tissue architectural and functional dysfunction as it restricts the delivery of oxygen and nutrients to organs and tissues. The extent of organ damage depends on the duration and degree of perfusion restriction, and on the organ’s biomolecular composition, levels of antioxidant defenses and metabolic rate (Wu M.Y. et al., 2018). Ischemia is a hallmark of several cardiovascular diseases, such as ischemic heart disease, peripheral artery disease and stroke (Johnson et al., 2019). During the ischemic period, cellular ATP stores are depleted by conversion into hypoxanthine and AMP. Xanthine dehydrogenase is converted into xanthine oxidase (XO), which converts hypoxanthine into uric acid (Johnson et al., 2019). From these processes, ROS are produced. Ischemia is also known to lead to endothelial dysfunction by increasing vascular permeability, and often leads to leukocyte infiltration and edema (Yang Q. et al., 2016). Albeit essential for preventing further tissue damage, the subsequent reperfusion process also happens to be deleterious to tissue due to oxygen overload, which leads to a massive ROS production by XO. Additionally, infiltrated leukocytes can lead to tissue damage by also secreting ROS themselves, as well as enzymes. Reactive oxygen species damage tissues by promoting lipid peroxidation and protein carbamylation (Verbrugge et al., 2015), which compromise the structure and function of cellular membranes, and lead to cell dysfunction and death via necrosis and/or apoptosis.

Caffeic acid, PLCA, CAPE, and nitro-CAPE derivatives have shown a very high potential for preventing and attenuating tissue damage caused by ischemia/reperfusion (I/R) injury in many organs *in vivo* and *in vitro*, including the myocardium (Ozer et al., 2004; Cagli et al., 2005; Huang et al., 2005; Parlakpinar et al., 2005; Tan et al., 2005; Özeren et al., 2005; Ince et al., 2006; Ho et al., 2013; Castellano et al., 2016), skeletal muscle (Cagli et al., 2005; Ozyurt et al., 2006, 2007; Andrade-Silva et al., 2009), brain (Irmak et al., 2001; Tsai et al., 2006; Altuǧ et al., 2008), spinal cord (Ilhan et al, 1999), liver (Feng et al., 2008; Saavedra-Lopes et al., 2008), kidney (Irmak et al., 2001; Gurel et al., 2004; Ozer et al., 2005a; Migliori et al., 2015), intestine (Koltuksuz et al., 1999; Sato et al., 2011; Teke et al., 2012, 2013), retina (Shi et al., 2010), ovary (Celli et al., 2004; Kart et al., 2009), testes (Koltuksuz et al., 2000; Eşrefoglu et al., 2005; Atik et al., 2006), and skin flaps (Aydogan et al., 2007; Yeǧin et al., 2020). Nearly all the studies conducted on the beneficial properties of these compounds on I/R injury agree that they are due to their potent ROS scavenging, antioxidant and anti-inflammatory activities. Regarding the cardiac I/R injury, these compounds normalize several biochemical changes associated with I/R injury. In particular, they increase CAT, GSH-Px and SOD activities, while reducing MDA levels, reflecting their antioxidant activity (Du et al., 2016; Li et al., 2018). They also decrease neutrophil infiltration, as observed by the decrease in MPO activity, further preventing oxidative injury and improves cardiac mechanical performance (Ku et al., 2016). In addition, these compounds suppress pro-apoptotic factors expression while upregulating anti-apoptotic factors (Du et al., 2016; Li et al., 2018). Pyrrolidinyl caffeamide in particular was shown to activate the PI3K-Akt pathway, leading to the activation of HO-1 enzyme, while stabilizing SOD and CAT activities (Ku et al., 2016). Pyrrolidinyl caffeamide also induces phosphorylation of protein kinase B (AKT) and AMP-activated protein kinase (AMPK), which leads to an increased expression of GLUT4 transporters, improving cellular energetics, and to the reduction of pro-apoptotic factors, improving cell survival (Lee et al., 2015a). Collectively, these effects contribute to a significant reduction in myocardial infarct size (Ozer et al., 2004; Cagli et al., 2005; Huang et al., 2005; Parlakpinar et al., 2005; Özeren et al., 2005; Ince et al., 2006; Ho et al., 2013; Castellano et al., 2016; Du et al., 2016; Li et al., 2018) and to an improvement in mechanical performance (Ku et al., 2016). Finally, the protective effect of CA and its derivatives over vascular endothelial cells facilitates reperfusion, leading to an increase in nutrient supply and waste product removal, as well as to the decrease in the migration of damaging mononuclear cells (Wang X. et al., 2006; Li et al., 2018).

### Anti-atherosclerotic Activity

Atherosclerosis is an inflammatory process consisting in the formation of a fatty plaque in the intimal layer of arteries, whose growth with time leads to progressive vascular lumen narrowing and to consequent perfusion reduction. The process begins with the uptake of LDLs carried in the bloodstream, a process that is stimulated by high levels of sheer stress on the endothelium. Upon accessing the intimal layer, LDLs are oxidized and taken up by macrophages which become foam cells and secrete cytokines to attract other leukocytes and growth factors. Consequently, VSM cells undergo considerable hypertrophy and hyperplasia. Over time, the fatty plaque is calcified, becomes hard, and is more susceptible to break and to acute vessel occlusion (Bergheanu et al., 2017).

Caffeic acid is described as an inhibitor of LDL oxidation, preventing atherosclerosis (Wang and Yang, 2012). Caffeic acid phenethyl ester has benefits for preventing the progression of atherosclerosis *in vitro* and in several animal models *in vivo*. In apolipoprotein E-deficient mice, oral CAPE administration (30 mg/kg for 12 weeks) ameliorates the atherosclerosis progress. It has been shown that CAPE inhibits NF-κB and related genes, such as TNF-α, interleukin 2, platelet-derived growth factor (PDGF) and E-selectin, in the aorta (Hishikawa et al., 2005) to, therefore, prevent the infiltration of leukocytes in the plaque milieu. Besides initiating atherosclerosis, LDL uptake is also associated with other hazardous changes in the cardiovascular system; for example, apoptosis of endothelial cells and increased expression of angiotensin II receptor, where the latter contributes to increase blood pressure (Sumners et al., 2015). In *in vitro* terms, pretreatment of human coronary artery endothelial cells with CAPE prevents the oxidized LDL (oxLDL) and TNF-α-mediated increase in angiotensin II receptor expression, which is thought to be achieved through NF-κB suppression (Li et al., 2000). In another study, CAPE has been found to inhibit oxLDL–induced apoptosis in human aortic endothelial cells, achieved by the action of the lectin-like endothelial receptor for oxLDL (Li and Mehta, 2000). Caffeic acid phenethyl ester also has beneficial effects on the smooth muscle layer. It inhibits angiotensin II, PDGF-induced proliferation and the migration of VSM cells, this last case via the activation of p38 MAPK, hypoxia inducible factor-1_α_ (HIF-1_α_), and HO-1 (Roos et al., 2011). Caffeic acid phenethyl ester inhibits the re-stenosis of injured carotid arteries of rats, probably due to the combination of NF-κB pathway inhibition, ROS scavenging, COX-2 inhibition and neointima apoptosis (Maffia et al., 2002). Diabetes is a marked factor that accelerates atherosclerosis. Caffeic acid phenethyl ester suppresses diabetes-induced atherosclerotic manifestations without affecting the developed hyperglycemia, particularly by preventing collagen deposition and, consequently, arterial stiffening (Hassan et al., 2014).

### Anti-angiogenic Activity

Angiogenesis refers to the tightly controlled and complex process of the formation of new blood vessels from pre-existing essential vessels for tissue development, regeneration and repair (Tahergorabi and Khazaei, 2012). Angiogenesis contributes to the growth of tumors, especially for the development of metastases. It is also a histological hallmark of atherosclerotic lesions, where it contributes to fatty plaque destabilization (Camaré et al., 2017) and occurs in peripheral arterial disease, where it contributes to tissue reperfusion (Inampudi et al., 2018). The angiogenesis process lies in a balance between promoting and suppressing factors. Angiogenesis is initiated when tissues sense a hypoxic environment by responding with the stabilization and translocation of transcription factor hypoxia-inducible factor 1-alpha (HIF1_α_) to the nucleus, where it induces angiogenic factors like vascular endothelium growth factor (VEGF) (Potente and Carmeliet, 2017). This factor is then released and binds to the VEGF2 receptor in neighboring endothelial cells to increase NOX activity, which leads to ROS production. This oxidizing environment activates many intracellular signaling pathways that lead to proliferation, migration and tube formation by endothelial cells (Fukai masuko and Nakamura, 2008). Vascular endothelium growth factor (VEGF) and other proangiogenic factors lead ROS themselves to increase VEGF secretion in tissues by reinforcing the neovascularization process (Kim and Byzova, 2014). Changes in the extracellular matrix, brought about by degrading enzymes (i.e., matrix metalloproteinase 9, MMP9) secreted by infiltrating neutrophils and by ROS, are also potent inducers of angiogenesis (Quintero-Fabián et al., 2019).

Caffeic acid phenethyl ester has inhibitory effects on various cancer cell lines, including lung cancer, melanoma, glioma and pancreatic cancer, colon cancer, breast cancer and neuroblastoma (Wu J. et al., 2011; Firat et al., 2019) both *in vitro* and *in vivo*. Caffeic acid, CAPE and synthetic derivative CADPE [3-(3,4-Dihydroxy-phenyl)-acrylic acid 2-(3,4-dihydroxy-phenyl)-ethyl ester], later found to be a naturally occurring compound also called teucrol, have shown anti-angiogenic effects on both cancer cell lines and the cell lines of diverse organs (Jung et al., 2007). The anti-angiogenic effects of CAPE have been demonstrated *in vitro* in chorioallantoic membrane (Song et al., 2002) and bovine capillary cell models (Wu J. et al., 2011), and in several models of cancer-bearing animals *in vivo*. These studies have established that CAPE’s antioxidant and anti-inflammatory properties are important for suppressing the angiogenesis process. (Ahn et al., 2009). Firstly, CAPE suppresses VEGF secretion both *in vitro* (cancer cell lines) and *in vivo* (tumor-bearing animals) (Liao et al., 2003; Paeng et al., 2015), an effect shared by CAPDE (Jung et al., 2007). This has been partly attributed to ROS scavenging and to the inhibition of the PI3K-Akt and HIF-1_α_ signaling pathways (Jung et al., 2007; Paeng et al., 2015). *In vitro*, CAPE also inhibits tube formation in HUVECs in a concentration-dependent manner (Liao et al., 2003; Ahn et al., 2009), besides showing antiproliferative activity of endothelial cells (Grunberger et al., 1988). Secondly, through its anti-inflammatory (LOX inhibition) and immunomodulatory (NF-κB suppression) properties, CAPE inhibits corneal neovascularization (Totan et al., 2001). Strikingly, CAPE has also been found to induce the activation of the NF-κB pathway and to promote VEGF expression in a dental pulp cell line (Kuramoto et al., 2019). This leads to the conclusion that the effect of CAPE on NF-κB and the angiogenic process is not as straightforward as previously believed, and differences in cell lines may explain this new found variability. Thirdly, CAPE is also able to interfere with the extracellular matrix modifications brought about by the leukocyte infiltration present in carcinogenesis by reducing the expression and inhibiting the activity of pro-angiogenic matrix metalloproteinases (Chung et al., 2004; El-Refaei and El-Naa, 2010; Wu S. et al., 2017), and by independently increasing the expression of angiostatic factors (El-Refaei and El-Naa, 2010) and shifting the equilibrium toward angiogenesis suppression.

## Conclusion

Caffeic acid and its derivatives display major actions on the cardiovascular system. Several compounds (CA, CAPE, and CAPA) display relevant vasorelaxant activity and attributed to diverse cellular mechanisms. Besides their vasorelaxant activity, CA and CAPE also lower heart rate and suppress the renin-angiotensin-aldosterone axis, which explains their blood pressure-lowering activity. However, there is a notable shortage of *in vivo* studies that center on exploring effects on perfusion. Furthermore, the pharmacokinetic profile of some CA derivatives is still lacking in both animals and human subjects, which should prompt further studies. Caffeic acid phenethyl ester is the most studied compound, and shows marked anti-atherosclerotic and anti-angiogenic effects, as well as protection against ischemia/reperfusion lesions, which is why it is the one with the highest potential highest potential for translation into clinical medicine.

## Author Contributions

HS and NL contributed literature research. HS contributed manuscript editing. Both authors contributed to the article and approved the submitted version.

## Conflict of Interest

The authors declare that the research was conducted in the absence of any commercial or financial relationships that could be construed as a potential conflict of interest.
